# Rotating-platform deep-dish total knee arthroplasty with restricted kinematic alignment: Five-year clinical and functional outcomes

**DOI:** 10.1051/sicotj/2025018

**Published:** 2025-06-06

**Authors:** Hannes Vermue, Guillaume Mesnard, Elvire Servien, Cécile Batailler, Sébastien Lustig

**Affiliations:** 1 Department of Orthopedic Surgery and Sport Medicine, Croix-Rousse Hospital, FIFA Medical Center of Excellence 69004 Lyon France; 2 Department of Orthopedic Surgery, Ghent University Hospital 9000 Ghent Belgium; 3 EA 7424, Interuniversity Laboratory of Human Movement Science, Université Lyon 1 69003 Lyon France; 4 Université de Lyon, Université Claude Bernard Lyon 1, IFSTTAR, LBMC UMR_T9406 69622 Lyon France

**Keywords:** Deep-dish, Rotating platform, TKA, Restricted kinematic alignment

## Abstract

*Introduction*: Total knee arthroplasty (TKA) utilizing deep-dish tibial inserts has gained interest due to its high congruency and enhanced stability. However, due to the advent of more personalized alignment philosophies, the combination of a rotating-platform deep-dish TKA design with restricted kinematic alignment (rKA) might improve patient satisfaction. Therefore, this study evaluated the five-year clinical and functional outcomes of rKA with a deep-dish TKA design. *Methods*: A retrospective analysis was conducted on patients who underwent primary TKA with a rotating-platform deep-dish design and rKA. Of 143 eligible patients, 123 completed five-year follow-up. Clinical and radiographic assessments included the five-year postoperative results: Knee Society Score (KSS), patient satisfaction, range of motion, coronal limb and implant alignment, postoperative complications and implant survivorship. Statistical analyses compared preoperative and postoperative outcomes with paired analyses. *Results*: Median KSS Knee and Function scores significantly improved from 70 (IQR 5) and 60 (IQR 26) preoperatively to 90 (IQR 20) and 93 (IQR 21) postoperatively (*p* < 0.001). Postoperative coronal alignment in this study encompassed a hip-knee-ankle angle was 178.1° ± 3.5, a Lateral Distal Femoral Angle of 89.9° ± 2.6, and a Medial Proximal Tibial Angle of 88.6° ± 2.2. At five years, 94% of patients were either satisfied or very satisfied. The revision-free survival rate was 98%. Periprosthetic joint infection and arthrofibrosis were the most common complications (1.6% for both groups separately), followed by aseptic loosening of a cementless femoral component (0.8%) and patellar dislocation (0.8%). *Discussion*: Rotating-platform deep-dish TKA with restricted kinematic alignment results in excellent functional outcomes, high patient satisfaction, and low complication rates at five-year follow-up. These findings support its viability as a surgical strategy, though long-term studies are warranted to assess implant durability and survivorship beyond 10 years.

## Introduction

Total knee arthroplasty (TKA) is a widely utilized surgical intervention aimed at alleviating pain and restoring function in patients with advanced knee arthritis. Among various TKA designs, deep-dish tibial inserts, characterized by their high congruency and enhanced anterior-posterior stability, are a viable option [1–3]. These inserts aim to optimize joint stability and improve postoperative outcomes without compromising mobility.

Deep-dish TKA designs have demonstrated equivalent short-term early postoperative functional outcomes when compared to standard posterior-stabilized (PS) or cruciate-retaining (CR) designs [2, 3]. For instance, Shatrov et al. reported no significant difference in clinical outcomes and range of motion (ROM) between patients undergoing condylar-stabilized deep-dish TKA and posterior-stabilized designs at short-term follow-up, emphasizing their utility as a versatile alternative [2]. Similarly, Stadler et al. highlighted the mid-term durability of cementless deep-dish TKA, showing excellent implant survival and negligible aseptic loosening at five years [4].

Similar to the different TKA designs possibilities there are several different strategies to position the femoral and tibial components [5]. Kinematic alignment focuses on restoring pre-arthritic alignment [6]. This method reduces the need for soft tissue releases and aims to preserve normal knee kinematics, which may potentially improve functional outcomes and patient satisfaction. However, there is an ongoing debate about the long-term implications of KA in TKA, particularly with respect to the difficulty restoring the pre-arthritic states and issues regarding patellofemoral tracking with implants designed for mechanical alignment [7–9]. As such, the concept of restricted kinematic alignment (rKA) has been proposed, which involves limiting the alignment within a narrower range of anatomical constraints. The restrictions in rKA could enhance the longevity of the implant and optimize its biomechanical behavior by more closely mimicking the natural joint mechanics [10]. While deep-dish TKA has demonstrated promising early outcomes, little is known about its performance in the context of restricted kinematic alignment over longer follow-up periods. This personalization of implant alignment along with the high congruency provided by the implant might further improve clinical outcomes and patient satisfaction in patients receiving TKA. As such, this study evaluates the five-year clinical and functional outcomes of rotating-platform deep-dish TKA with restricted kinematic alignment.

## Materials and methods

This retrospective study was conducted at a single tertiary arthroplasty center, analyzing patients who underwent deep-dish total knee arthroplasty for end-stage osteoarthritis in 2018 and 2019.

Inclusion criteria were: all patients receiving primary TKA for end-stage osteoarthritis, use of a rotating-platform deep-dish TKA design, and minimum postoperative follow-up of five years. Exclusion criteria were: collateral ligamentous insufficiency, severe extra-articular deformities (>10°) requiring corrective osteotomies, post-traumatic arthritis with substantial bone loss, and history of infection, incomplete radiographic and clinical follow-up. Ethics approval was obtained from our institutional ethics committee (ICP-D106.02). Written informed consent was obtained from all patients and/or families. Of the 143 patients eligible to the in- and exclusion criteria, 14 were lost-to follow-up in the hospital prior to completion of the 5-year follow-up. Five patients died during the follow-up period. The demographics of all patients included in the study can be found in Table 1.

**Table 1:** Patient demographics.

Variable	Result
Age [years]	71.3 ± 8.6
Sex [%W/%M]	69/31
Side [%L/%R]	43/57
BMI [kg/m^2^]	30.0 ± 6.0
Charnley	A (40%), B1 (51%), C1 (1%), C3 (7%)
Devane	3 (36%), 4 (62%), 5 (2%)
Follow-up [years]	5 ± 1
Surgical time [min]	78.1 ± 16.2

W = Women, M = Men, L = Left, R = Right.

### Surgical technique

All procedures were performed by a fellowship-trained arthroplasty surgeon using a standardized approach, with a rotating-platform deep-dish design (Score 2, Amplitude, and Valence, France). Tourniquet was not used throughout the study. In case of varus osteoarthritis, a subvastus approach was performed, whereas a lateral parapatellar approach was performed in case of valgus osteoarthritis (Hip-Knee-Ankle angle (HKA) > 183°). A restricted kinematic alignment philosophy was used to perform the bony cuts and position the femoral and tibial implants [11]. The tibial and, in case of patellar resurfacing, the patellar components were cemented. The femoral component was either cemented or cementless, based on the bone quality of the patient, as assessed preoperatively on radiographs or intraoperatively. In total 10% of patients received a cementless femoral implant. Local infiltration analgesia with a combination of ropivacaine, epinephrine, and clonidine was administered intraoperatively. Patellar resurfacing was performed selectively, based on intraoperative cartilage quality, Iwano classification grade 2 or higher, and preoperative symptoms.

### Postoperative management

All patients underwent the same postoperative rehabilitation protocol. Early mobilization was encouraged, with weight-bearing as tolerated starting on postoperative day one. Physiotherapy included range-of-motion exercises, quadriceps strengthening, and gait training. Patients were discharged when they achieved adequate pain control, independent mobility, and knee flexion of at least 90°.

### Outcomes assessed

Patients received routine follow-up at 2 months, 1 year and 5 years. In case of any irregularity or patient concern, earlier follow-up was scheduled. The following variables were collected: Demographic data, including age, sex, body mass index (BMI), Charnley classification, and Devane classification [12, 13]. Clinical and functional assessment was evaluated using the Knee Society Score (KSS), including both the Knee and Function subscales (0: worse, 100: best) [14]. As well, the HKA, Mechanical Medial Proximal Tibial Angle (mMPTA), Mechanical Lateral Distal Femoral Angle (mLDFA), Blackburn-Peel index, patellar tilt and patellar mediolateral position were analyzed on full leg standing radiographs, anteroposterior (AP), lateral and axial knee views. Complications were recorded during follow-up. More specifically, surgical site infection or periprosthetic joint infection (PJI) were diagnosed based on the Musculoskeletal Infection Society criteria [15]. Other complications such as hematoma, aseptic loosening, periprosthetic fracture, arthrofibrosis, instability, and patellar issues were defined based current literature [16, 17]. Survivorship analysis was based on revision TKA for any cause.

### Statistical analysis

SPSS (Version 23, IBM, USA) was used for all statistical analyses. Shapiro-Wilk tests were used to verify normality of the data, which was visually verified with boxplots. Continuous variables are presented as means and standard deviations (SD) in the case of parametric data, and median with interquartile range (IQR) in case of non-parametric data. Comparisons between preoperative and postoperative outcomes were analyzed using paired t-tests in case of parametric data, whereas otherwise the non-parametric Wilcoxon test was used. Categorical variables were reported as frequencies and percentages. Kaplan-Meier analysis was used to evaluate implant survivorship. A *p*-value of <0.05 was considered statistically significant.

An a priori sample size calculation was performed with GPower (Version 3.1, Heinrich Heine University, Düsseldorf, Germany) based on a minimally clinical important difference in KSS Knee score of 6.6 (SD 24.0) with a power of 80% and an alpha level of 0.05 [18]. This analysis determined that 106 subjects were required to achieve sufficient statistical power.

## Results

At 5 years of follow-up 94% were either satisfied (49%) or very satisfied (45%) with their postoperative result. Six percent of patients were dissatisfied at 5 years postoperative. The median KSS Knee scores improved from 70 (IQR 5) preoperatively to 90 (IQR 20) postoperatively (*p* < 0.001). Similarly, KSS Function scores increased from 60 (IQR 26) to 93 (IQR 21) postoperatively (*p* < 0.001).

As expected with a restricted kinematic alignment technique, the HKA was corrected to 177 (IQR 5.8) postoperatively. without any statistically significant difference for mLDFA (89.0 ± 3.0 vs 89.9 ± 2.6; *p* = 0.57; Table 2). The mMPTA did differ between the pre- and postoperative situation (86.0 ± 3.6 vs 88.6 ± 2.2; *p* < 0.001). Blackburn Peel ratio increased slightly postoperatively from 0.65 ± 0.13 to 0.69 ± 0.16 (*p* = 0.023). On the axial radiographs, patellar position was central in 92%, although some tilt was observed in 5% and subluxation in 3% of cases.

**Table 2:** Comparison between the preoperative and postoperative radiological parameters, range of motion and KSS scores.

Variable	Preoperative	Postoperative	*p*-value
HKA [degrees]	172.0 (IQR 4.8)	177 (IQR 5.8)	<0.001
mLDFA [degrees]	89.0 ± 3.0	89.9 ± 2.6	0.57
mMPTA [degrees]	86.0 ± 3.6	88.6 ± 2.2	<0.001
Blackburn Peel	0.65 ± 0.13	0.69 ± 0.16	0.023
Range of motion [degrees]	120 (IQR 10)	128 (IQR 10)	0.254
KSS knee	70 (IQR 5)	90 (IQR 20)	<0.001
KSS function	60 (IQR 26)	93 (IQR 21)	<0.001

HKA = Hip-Knee-Ankle axis, mLDFA = mechanical Lateral Distal Femoral Angle, mMPTA = mechanical Medial Proximal Tibial Angle.

**Figure 1:**
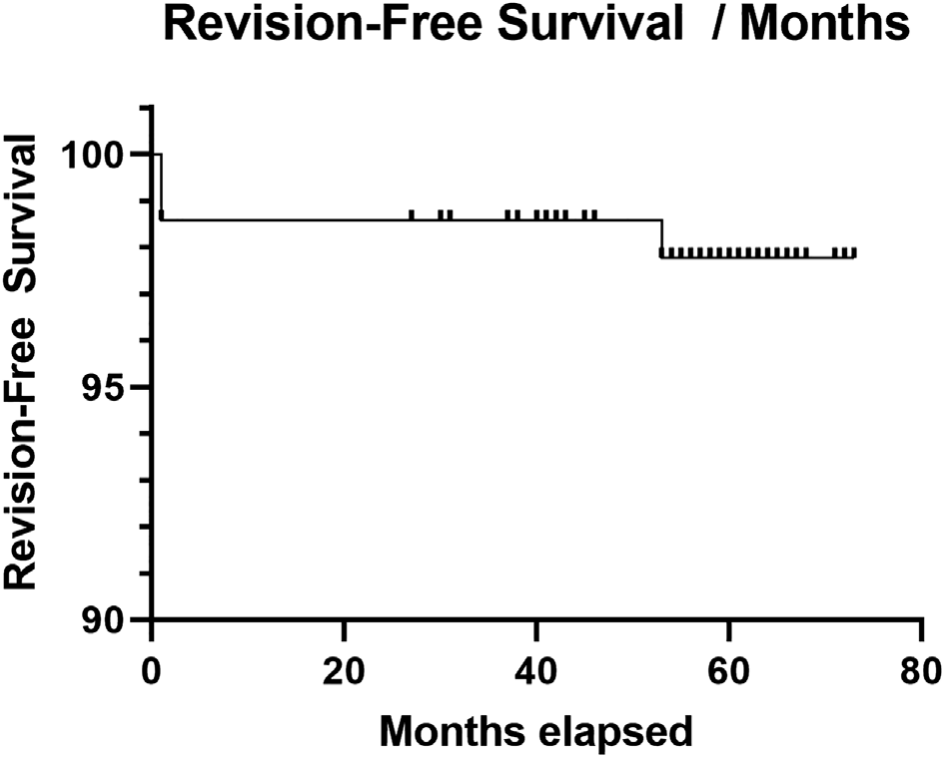
Kaplan Meier curve demonstrating revision-free survival rates over the follow-up period (Months).

In total there were 5 patients who suffered complications following their TKA (Table 3). PJI and arthrofibrosis were most common (1.6% for both groups separately), followed by aseptic loosening of a cementless femoral component (0.8%) and patellar dislocation (0.8%). The revision-free survival, which was 98% at 5 years, can be seen in Figure 1.

**Table 3:** Overview of the complications observed during the 5 years of follow-up in this study.

Complication	Treatment
PJI	DAIR and due to recurrence 2-stage revision
Aseptic loosening	Revision TKA
Patellar dislocation	MPFL reconstruction
Arthrofibrosis	Mobilization under Anesthesia
Arthrofibrosis	Arthroscopic arthrolysis
PJI	2-stage revision

Same patient.

## Discussion

In current literature there is high focus on personalization in TKA, aiming to provide superior clinical outcomes and satisfaction. The findings of this study adds to that body of evidence demonstrating that deep-dish TKA yields excellent clinical outcomes and high patient satisfaction at a five-year follow-up, together with high implant survival. The insights gained from this study may guide future practices in TKA, particularly in the context of refining the combination between alignment strategies and implant design in an attempt to optimize patient satisfaction and clinical function.

Despite the strong clinical outcomes observed, our study has several limitations that must be acknowledged. First, its retrospective design introduces potential recall bias, as patient-reported satisfaction and function scores rely on subjective recollection rather than standardized prospective assessments. Second, while this study was conducted at a single center with high volume surgeons, the generalizability of our results to other institutions and patient populations might be limited. Multi-center trials with diverse cohorts would help confirm whether these outcomes are replicable across different surgical teams and healthcare settings. Third, no comparative analysis was performed analyzing differences between a different alignment philosophy or implant design. However, the discussion below aims to thoroughly assess the results of this study in light of current literature (Table 4).

**Table 4:** Overview of the studies currently reporting clinical results or complications on deep-dish TKA.

Author	Year	*n*	Age	Implant	FU [years]	Complications	Clinical results
Laskin et al.	2000	66	71	Genesis II (Smith & Nephew)	NA	1 PE	KSS knee: 94 (82–100)
						1 exfoliative dermatitis	KSS function: 55 (45–85)
							ROM: 117 (95–135)
							Patellar tilt: 5%
Czekaj et al.	2017	154	75 ± 7	Score (Amplitude)	5	3 Arthrofibrosis	KSS knee: 94 ± 7
						2 Periprosthetic fracture	KSS function: 82 ± 20
						1 SSI & 1 PJI	ROM: 114 ±10
						1 Anterior knee pain	
						1 Aseptic loosening	
Stirling et al.	2019	54	69 ± 9	Triathlon (Stryker)	1	/	OKS: 33 ± 10
							EQ-5D: 0.7 ± 0.3
							Pain VAS: 71 ± 28
							Satisfaction: 83%
Shatrov et al.	2022	167	72 ± 8	Score II (Amplitude)	3	1 Patella resurfacing	KSS knee: 86 ± 14
						1 PJI	KSS function: 84 ± 13
						1 Aseptic loosening	ROM: 119 ± 5
							Satisfaction: 17%
Stadler et al.	2022	91	67 ± 7	Vanguard (Zimmer Biomet)	5	1 PJI (DAIR)	KSS knee: approximately 94
						1 Patella resurfacing	KSS function: approximately 90
						1 Periprosthetic fracture	KOOS: 85
						1 Wound dehiscence	ROM: 118 ± 10
						1 Avulsion MCL	
						1 Artrofibrosis	
Lefèvre et al.	2022	106	69 (IQR 64–76)	KneeTec (Corin)	1	2 SSI	KSS knee: 92 (IQR 77–97)
						1 Insert spinout	KSS function: 88 (IQR 70–100)
							OKS: 40 (IQR 33–43)
							ROM: 120 (IQR 110–130)
							Patellar tilt/subluxated/dislocated: 3%/10%/2%

FU = Follow-up; NA = Not available; PE = Pulmonary Embolism; KSS = Knee Society Score; ROM = Range of Motion; SSI = Surgical Site Infection; PJI = Periprosthetic Joint Infection; OKS = Oxford Knee Score; VAS = Visual Analogue Scale; DAIR = Debridement and Implant Retention; KOOS = Knee Injury and Osteoarthritis Outcome Score; MCL = Medial Collateral Ligament.

The KSS Knee and KSS Function in this study were similar to scores reported in other studies assessing deep-dish TKA outcomes (Table 4). For example, several studies found that patients receiving deep-dish TKA demonstrated mean KSS Knee values between 85.5 and 94, depending on the specific implant system used [1, 2, 4, 19]. A retrospective study by Lefèvre et al. found that deep-dish designs showed median postoperative KSS scores of 91.5, which was not significantly different from a PS design [20]. Similarly, Shatrov et al. could not demonstrate any difference in clinical outcomes between their deep-dish cohort and matched CR cohort at 3-years postoperatively [2]. While deep-dish TKA theoretically could be associated with a risk of limited flexion due to its constraint, our findings suggest that functional outcomes remain excellent, further supporting its continued use in clinical practice [21]. Of course, these slight similarities and differences should be interpreted considering several factors, for example the study location, patient demographics, surgical protocol, postoperative pain management and rehabilitation.

A remarkable aspect of our findings is the 94% satisfaction rate, which is higher than that reported in many historical studies [22], as well as on deep-dish TKA (Table 4) [2, 3, 20]. One of the studies on deep-dish TKA by Lefèvre et al. was similar to our results with satisfaction rates around 94% at five years postoperatively, which is in contrast to the study of Shatrov et al. and Stirling et al., which has stated a dissatisfaction rate of 17% in their deep-dish TKA cohort at 3 and 1 years postoperatively, respectively [2, 3]. The study by Stirling et al. used a fixed bearing design, although there is no clear superiority of either fixed- or mobile bearing designs in current literature. However, an upward trend in satisfaction might be seen with more recently published studies, in particular when modern surgical, anesthesiology, and rehabilitation principles are applied.

One potential reason for the success of deep-dish TKA in our study is the use of restricted kinematic alignment, which appears to offer an optimal balance between mechanical and unrestricted kinematic alignment [11]. While traditional mechanical alignment ensures standardized component positioning, it does not always respect individual patient anatomy, which could lead to suboptimal kinematics and eventually, clinical outcomes [23, 24].

Recent studies have highlighted the benefits of restricted kinematic alignment. A study by Vendittoli et al. found that restricted kinematic alignment resulted in improved gait mechanics, quadriceps function, and patient-reported outcomes compared to strict mechanical alignment [11]. However, comparative trials evaluating alignment techniques with rotating-platform deep-dish TKA are currently lacking. Our study adds to the growing body of evidence by demonstrating that restricted kinematic alignment allows for excellent clinical outcomes and patient satisfaction in deep-dish TKA, further supporting its clinical relevance.

In general, the complication rate in this study was similar to other reported studies on rotating-platform TKA [25]. There was no tibial loosening seen, which theoretically might be a concern with the highly conforming insert. The rotating platform seems to protect the tibial component from early loosening, similar to the study by Stadler et al. [4]. While our study demonstrates excellent mid-term results, the long-term durability of deep-dish TKA remains an important question. Some prior reports have suggested that while deep-dish TKA provides good stability, the increased conformity may limit femoral rollback and reduce flexion capacity compared to more traditional designs [21]. Future research should focus on long-term survivorship, component wear, and revision rates beyond 10 years to determine whether these promising mid-term outcomes translate into durable, long-term success.

## Conclusion

This study demonstrates that a rotating-platform deep-dish TKA with restricted kinematic alignment provides excellent mid-term outcomes, with high patient satisfaction, significant functional improvements, and a 98% revision-free survival at five years. Multicenter trials and extended follow-up beyond 10 years will help determine whether implant loosening might be an issue.

## Data Availability

The data that support the findings of this study are available from the corresponding author upon reasonable request.
